# Tampering of Viruses and Bacteria with Host DNA Repair: Implications for Cellular Transformation

**DOI:** 10.3390/cancers13020241

**Published:** 2021-01-11

**Authors:** Francesca Benedetti, Sabrina Curreli, Robert C. Gallo, Davide Zella

**Affiliations:** 1 Institute of Human Virology and Global Virus Network Center, Department of Biochemistry and Molecular Biology, University of Maryland School of Medicine, Baltimore, MD 21201, USA; FBenedetti@ihv.umaryland.edu; 2 Institute of Human Virology and Global Virus Network Center, Department of Medicine, University of Maryland School of Medicine, Baltimore, MD 21201, USA; SCurreli@ihv.umaryland.edu (S.C.); rgallo@ihv.umaryland.edu (R.C.G.)

**Keywords:** DNA repair, DNA damage, carcinogenesis, bacteria, viruses, cellular pathways

## Abstract

**Simple Summary:**

A large number of DNA damages occur per cell every day due to several causes, including viral and bacterial infections. The majority of them are repaired, but in some cases the repair processes are not totally efficient due to viral and bacterial proteins interferences with the host cellular machineries. Not repaired damages increase mutations, that ultimately cause genomic instability that increases the risk of cancer. The increased consideration of the role of DNA damage and repair in tumorigenesis has implications for the prevention and treatment of cancer. Our review provides a framework to better understand the common role played by some viruses and bacteria in cellular transformation.

**Abstract:**

A reduced ability to properly repair DNA is linked to a variety of human diseases, which in almost all cases is associated with an increased probability of the development of cellular transformation and cancer. DNA damage, that ultimately can lead to mutations and genomic instability, is due to many factors, such as oxidative stress, metabolic disorders, viral and microbial pathogens, excess cellular proliferation and chemical factors. In this review, we examine the evidence connecting DNA damage and the mechanisms that viruses and bacteria have evolved to hamper the pathways dedicated to maintaining the integrity of genetic information, thus affecting the ability of their hosts to repair the damage(s). Uncovering new links between these important aspects of cancer biology might lead to the development of new targeted therapies in DNA-repair deficient cancers and improving the efficacy of existing therapies. Here we provide a comprehensive summary detailing the major mechanisms that viruses and bacteria associated with cancer employ to interfere with mechanisms of DNA repair. Comparing these mechanisms could ultimately help provide a common framework to better understand how certain microorganisms are involved in cellular transformation.

## 1. Introduction

The series of events that lead to cellular transformation may be linked to the altered activity of different proteins which belong to several cellular pathways. A comprehensive review of them is outside the scope of this report. This review focuses only on the mechanisms that viruses and bacteria have evolved to hamper the pathways dedicated to maintaining the integrity of the genetic information, thereby impairing the repair of DNA damage(s). Though genetic changes are essential for adaptation and evolution, repairing the inevitable random lesions that ensue is essential for avoiding cellular transformation. This delicate balance is assured by complex and accurate mechanisms which have evolved in eukaryotic cells. Thus, a number of proteins act in concert for both replicating DNA and guiding its proofreading to avoid the transfer of harmful mutations to the daughter cells. The detrimental mutations must be either corrected or the cell eliminated to avoid cell transformation.

Among the vast majority of the thousands of changes that happen every day in the DNA of a human cell due to mistakes or environmental factors—like heat, radiation of various sorts, or exposure to substances—only a few are fixed as permanent mutations in the genome. The majority of these mutations are immediately and effectively corrected by the coordinated intervention of a number of proteins encompassing a large portion of the genome and collectively working together in organized pathways to ensure proper DNA repair. Some of these pathways are conserved in their overall organization, and many of the genes that encode them were first recognized in bacteria, by isolating and characterizing specific mutants with reduced or altered viability when cultured in the presence of DNA-damaging agents. In humans, disorders of DNA repair are linked to a variety of human diseases [1,2], and in almost all cases they are associated with the development of cellular transformation and cancer.

Either by themselves or in combination with other co-factors, certain viruses and bacteria are able to cause cancers by affecting important cellular pathways. Considering the delicate balance between DNA repair, proliferation, and apoptosis it is clear that perturbation of this balance is likely to result in cellular transformation. Here, we review several mechanisms whereby some proteins of certain viruses and bacteria interact with key players of cellular DNA repair pathways, eventually hampering the appropriate response and preventing the cells from undergoing apoptosis.

## 2. DNA Repair and Cellular Transformation

Due to the continuous exposure to different sources of damaging agents, DNA relies on multiple complex and synchronized repair pathways to maintain genomic integrity [1,2]. Inability to properly and timely repair DNA damage frequently originates in a number of genomic aberrations, from minor point mutations, to chromosomal translocations, gain or loss of chromosomal segments, and in some cases to major damage like loss of entire chromosomes [3,4,5,6]. Not surprisingly, in some cases one or more of these genomic changes eventually result in aberrant cellular functions, causing tumor initiation [6,7,8]. Beside its role in tumor initiation, altered DNA repair activity and fidelity is implicated in tumor development, progression, aggressiveness, and response to specific therapies. Additionally, the tumor micro-environment has several characteristics that increase DNA damage, namely high levels of oxidative and replication stress, and reduced/absent DNA damage–induced cell-cycle checkpoints, in some cases compounded by the concomitant loss of one or more DNA repair pathway [9].

The DNA damage response (DDR) refers to a number of proteins that first scan and detect DNA damage (sensors) and then are involved in its repair. DNA double-strand breaks (DSBs) are genomic lesions that may lead to carcinogenesis if left unrepaired or not properly repaired. The major pathways participating in DNA damage detection and repair are: (i) the nucleotide excision repair (NER) pathway, involved in eliminating bulky adducts; (ii) the mismatch repair (MMR) involved in recognition of errors occurring during DNA replication and homologous recombination (HR) or non-homologous end joining (NHEJ), eventually recognizing and repairing DSBs; (iii) the base excision repair (BER) pathways, involved in oxidative damage to DNA bases and single-strand breaks (SSBs) [10,11,12,13,14].

The choice of which pathway to use is not exclusive and in certain cases they can overlap, depending on the type of DNA damage [15,16]. An important difference is that while BER, NER, MMR, and HR pathways are essentially error-free, on the contrary NHEJ introduces some errors and may be responsible for mutated DNA sequences [17].

Several pathways are involved in the different mechanisms of DDR signaling and repair, notably the PI3K-related protein kinases ataxia telangiectasia (AT) mutated (ATM), ATM and RAD3-related (ATR), and DNA-dependent protein kinase (DNA-PK) pathways [18,19,20,21,22]. These proteins are Ser/Thr kinases that controls cell-cycle checkpoint, DNA replication, DNA repair, and apoptosis in response to DNA-damaging agents.

In a cell, ATM is typically present as inactive homodimers, which become active ATM monomers upon autophosphorylation of S1981 in response to DSBs. A polyprotein complex then is formed, since these active monomers are recruited with the MRE11–RAD50–NBS1 (MRN) to sites of DSBs and in turn direct DSB repair (DSBR) [20,23]. Of note, MRN serves not only a substrate for ATM kinase, but is necessary for complete activation and functionality of ATM itself. Moreover, ATM recruits to DSB two of its substrates, phosphorylated H2A histone family member X (also referred to as γH2AX) and Mediator of DNA Damage Checkpoint 1 (MDC1), together with the additional proteins RNF8 and RNF168, two histone-directed ubiquitin ligases [24]. The repair of DSBs by ATM is realized through homologous recombination (HR) repair. To this regard, phosphorylation of TIF1b/KAP1 by ATM allows 53BP1 and BRCA1 to be recruited to the proper DNA region [25]. ATM is also involved in p53-dependent G1/S cell-cycle checkpoint control, and in intra-S phase and G2/M checkpoint control [23]. To further underline the importance of ATM, its loss of function in AT, an autosomal recessive disorder characterized by progressive cerebellar ataxia, is associated with neurodegeneration and a predisposition to cancer [23,26]. The ATR pathway is similar to ATM, and it is required for the response elicited by both DDR and cellular pathways involved in DNA repair in response to DNA damaging agents. The ATR pathway organizes and synchronizes the cellular response to ssDNA, and it controls DNA replication in the S phase at stalled replication forks [27].

Recruitment of ATR to replication sites or sites of damage is achieved by a multi-step process, multi-protein complex that results in activation of ATR, through the ATR-interacting protein (ATRIP). ATRIP binds directly to RPA70 and then the RAD9–RAD1–HUS1 (9-1-1) replicative sliding-clamp complex associates with dsDNA junctions adjacent to RPA-loaded ssDNA. Finally, the BRCT-repeat protein TOPBP1 is recruited to the ATR–ATRIP complex. Eventually, the ATR-activation domain of TOPBP1 enables substrate binding and the activation of ATR kinase [27].

CHK1 is probably the best-characterized effector of ATR, and it regulates the G2/M checkpoint mainly by controlling CDC25 phosphatases activity. In fact, phosphorylation of CDC25 proteins inhibits CDC25 phosphatase of CDK1 thus blocking cellular entry into mitosis and DNA replication origin firing (multiple origins activated at different times in S phase) triggered by replication stress [27].

DNA-PK is a very important player in DSBR by tightly controlling the process of NHEJ [28,29]. DNA-PK is composed by DNA-PKcs, a large catalytic subunit, and Ku70 and Ku86, two smaller regulatory subunits. By recognizing and binding to DSBs, the Ku complex is able to recruit and stabilize the interaction of DNA-PKcs with DNA. A homodimer composed of two DNA-PKcs molecules brings DNA ends together in a synaptic complex, allowing the recruitment of the DNA ligase IV–XRCC4 complex. This complex in turn facilitates the sealing of the damaged DNA ends [30,31,32].

Cancer cells show genomic alterations consisting of various types of mutations and aberrant expression of genes involved in DNA repair responses that induce genome instability, promote carcinogenesis steps, and favor cancer progression. Indeed, due to their widespread presence, these deficiencies in DNA repair are been exploited as targets for most recent cancer therapies. These therapies are not within the main scope of this review, though below we mention some of them. For example, “homologous recombination repair defect” is a major clinical target of BRCA1/2 mutations with inhibitors of poly(ADP-ribose) polymerase (PARP), involved in DNA repair, shown to cause synthetic lethality [33,34]. In addition, some recent data indicate that DNA repair could be an important biomarker of immune checkpoint blockade (ICB) response [35,36]. Tumors with deficiency in MMR have high response rates to ICB, and the use of the anti–PD-1 agent pembrolizumab has been very recently approved by the FDA in patients with MMRd solid tumors or refractory MSI-H (microsatellite instability-high). A number of ongoing studies are now actively evaluating the possibility of using ICB agents to treat several types of tumors with DNA repair–deficiency, including ones with mutations in *BRCA1/2* or *POLE* [37,38,39].

These studies are exploring uncharted territories, and they will need the support of data generated both in vitro and in vivo to explore and understand the complexities of the interaction between DNA damage, reductions in the activity of DNA-repair pathways and immune-modulating agents [40].

Finally, it should be noted that a reduced activity of the pathways involved in DNA repair renders tumor cells more sensitive to therapy. On the downside, non-tumor cells getting DNA damage from therapy’s side effects could generate additional tumors. For this reason, DNA repair potential has to be taken into account when employing chemo- and/or radiotherapy treatments, suggesting that in certain cases a personalized therapy based on the patient’s cell DNA repair function is indicated [41].

## 3. Viruses–Host Interaction and Effects on Host’s DNA Repair

Due to their size (from a few Kb to about 200Kb), viral genomes have reduced coding ability. For this reason, they rely on cellular proteins and interfere with cellular proliferation and DNA repair processes to complete their life cycles and increase the production of viral particles [42]. In this regard, certain viruses activate separate cellular pathways encompassing DDR kinases, but these pathways differ from signaling commonly triggered in response to genomic DNA damage. Consequently, sometimes it is not easy to recognize whether a DDR signaling that is stimulated during infection results from a proper cellular response to viral replication or whether it is a response prompted by the virus to stimulate its own replication. In addition, activation of some proteins of the DDR pathways may negatively affect viral replication, indicating that they have antiviral properties. On the other hand, sometimes activation of DDR signaling facilitates viral misuse of essential cellular functions [42]. Finally, it some cases it seems that the outcome of DDR signaling in response to cellular DNA damage is cell cycle dependent [43]. Here we review the well-known associations of certain DNA and RNA viruses with cancers, namely HTLV-1, HPV, HBV, HCV, EBV, and KSHV (Table 1). This review will focus mostly on the first four, which are the most oncogenic ones. In general, these viruses promote oncogenesis by a multi-steps process affecting genes implicated in several cellular pathways, either involved in tumor initiation and/or acting at later stages supporting tumor promotion and spreading, like (but not limited to) regulation of cell proliferation, apoptosis, and senescence. Indeed, different viruses can affect different stages of tumor formation, even though the process is not virus-specific. Though not within the scope of this review, for the most well-known oncogenic viruses we will shortly summarize the major know mechanisms whereby specific viral proteins promote different stages of tumor development and we will only focus on post-infection events. In addition, we will highlight the viral protein(s) that specifically interact with and dysregulate components of different cellular pathways of DNA repair which promote cellular transformation.

The cell cycle status at the time of DNA damage determines the effects of DDR signaling by influencing viral replication and its susceptibility to manipulation by virus infection [43]. For example, the tumor suppressor pRb is often a target of viral oncoproteins, and this allows the unbound of E2F transcription factor to stimulate cell cycle progression and entry into S phase, thus improving the ability of the virus to access the cellular replication machinery [44]. Activating DDR signaling in another phase of the cell cycle, namely G2, can be employed as an alternative strategy, and also this inference of cell cycle functions, DNA damage signaling, and repair pathways eventually may result in modification/damage of host genome integrity.

### 3.1. HTLV-1

We note that among human RNA tumor viruses, the first known human retrovirus human T-cell leukemia virus type1 (HTLV-1) has been the only one most convincingly linked to a cancer, adult T-cell leukemia/lymphoma (ATL) and causing transformation without requiring any know cofactor [97,98,99,100]. The World Health Organization classifies it as one of the most potent oncogenic agents. ATL onset has a very long incubation time post-infection, and about 5% of cases develop leukemia [101]. The area with the highest HTLV-1 prevalence is Southern Japan, and about 15 to 20 million people are infected worldwide [102]. The virus is mainly spread through sexual contact and parenterally (for example blood transfusions, needles, breastfeeding, etc.). HTLV-1 is also found in HTLV-1-associated myelopathy/tropical spastic paraparesis (HAM/TSP), which is a rare disease that is thought to result from immunological aspects of host–virus interactions [103,104].

The genome of HTLV-1 (about 9Kb) contains the classical retroviral genes, i.e., gag, pol, and env, plus a region designated pX, which encodes for several regulatory proteins (including Tax and Rex). Both proteins are necessary for viral replication, and Tax acts as a transcriptional trans-activator affecting viral and cellular gene expression, increases cell proliferation and it is considered the major transforming protein of HTLV-1, although the precise molecular mechanism(s) originating chromosomal abnormalities in HTLV-I infected cells is not completely understood yet [105,106,107]. HTLV-1 integration has been demonstrated into the host genome in ATL patients [108]. By some authors, this has been taken as experimental evidence that HTLV-1 could cause insertional mutagenesis, though this interpretation is still open to debate and needs further data to be conclusively established [100,109,110,111]. Tax-mediated cellular transformation is also partially the result of suppression of DNA repair pathways that in turn increase the frequency of genomic mutations, and certain types of aberrations—including duplications, deletions, translocations, rearrangements, and aneuploidy—are commonly observed in ATL cells. Tax can undergo posttranslational modifications, namely acetylation, phosphorylation, ubiquitination, and sumoylation [105] and it does not bind directly to DNA, instead binding and modulating the activity of several transcription factors [112]. Tax expression may be sufficient for the immortalization of human T lymphocytes in vitro but it seems that in certain cases other factors may be necessary, as indicated by the fact that while Tax expression was necessary for growth of the primary T cells, addition of IL-2 was also required to drive the T cells into cell cycle progression [113,114].

Another important protein which exhibits a variety of oncogenic properties is the HTLV-1 Basic leucine-Zipper factor (HBZ) HBZ, which is regulated by the 3′ LTR and constitutively expressed in ATL patients [115]. HBZ has been shown to affect a number of cellular functions [116] and to induce T cell lymphoma in vivo [117,118].

#### Effect of HTLV-1 on DNA Repair

Multi-step genetic passages have been described in the immortalization and transformation process of an HTLV-I infected cell [119]. Indeed, a number of studies characterizing chromosomal abnormalities in ATL patients and in HTLV-I infected/transformed cells have established several clonal chromosomal abnormalities with complex karyotypes in both numbers and structures, but no clear association with a definite karyotypic abnormalities has been found in ATL cells [120]. The effects of Tax on DNA double-strand break repair are still poorly understood, though studies using the gene array technique showed that Tax expressing cells have reduced levels of Ku80 mRNA [45], a key component of the DNA dependent protein kinase complex (DNA-PK) which acts as sensor and regulator element of DSB repair. In other studies, Tax has been shown to constitutively activate DNA-PK and to attenuate ATM signaling in response to DNA damage [46,47]. Taken together, these data suggest that Tax, by affecting DSB repair, might effectively promote mutagenesis [46,121]. In addition to DNA-PK, Tax binds and sequesters other factors in DNA damage-independent repair foci, including BRCA1 and MDC1. In particular, foci localization is determined by the N-terminal region of Tax, while its C-terminal half is critical for MDC1 binding and recruitment of other response factors [48]. Of note, HTLV-I Tax induces DNA DSBs during DNA replication, thus promoting genetic instability while also activating the NF-κB pathway to eventually inhibit the homologous recombination (HR) DNA repair pathway [49]. In addition, Tax suppresses BER and NER by altering the levels of key cellular factors involved in these pathways, including DNA polymerase β and PCNA, respectively [50,51,52,53]. It thus appears that Tax suppresses NER while allowing replication of damaged DNA and consequent introduction of mutations in the genome of cells infected by HTLV-I, and this could help to explain the observation that Tax expression has been correlated to a higher frequency of mutations observed in these cells [122]. It is known that an increased accumulation of spontaneous mutations within cells is associated with inactivation of DNA MMR, leading to microsatellite instability. Tax expression may also seem to interfere and reduce the activity of the MMR pathway, as suggested by data showing a reduction or loss in expression of some MMR genes and microsatellite instability in primary leukemic cells from ATL patients and the ability of Tax to repress pol β expression [123,124,125]. In addition, Tax inactivates transcription of telomerase, and this in turn can leave unprotected DNA ends that are more susceptible to recombination and translocation events, in turn leading to karyotypic abnormalities [54]. p30, another HTLV-1 protein modulating viral replication and pathogenesis [126], has also been shown to inhibit the conservative homologous recombination (HR) DNA repair by targeting the MRE11/RAD50/NBS1 complex, and that in turn promotes the error-prone NHEJ DNA-repair pathway. It is thus likely that HTLV-1 p30 may enable the accumulation of DNA mutations in the infected cell and the possibility of transformation [55]. Finally, HBZ is another protein of HTLV-1 that is responsible for interfering with DNA repair, in particular it reduces repairs of the double-stranded DNA breaks via non-homologous end joining pathway [56]. To exert this effect, HBZ interacts with two important members of the NHEJ core machinery, namely Ku70 and Ku80 [56].

### 3.2. HPV

Human papillomaviruses (HPVs) are non-enveloped dsDNA viruses belonging to the Papillomaviridae family, with a short circular genome of about 8 kbp. HPVs targets undifferentiated cells in the basal layer of stratified epithelium and upon infection their genome is maintained in an episomal form. The HPV replication cycle is closely associated to the differentiation status of the epithelium, and the different viable steps of viral genome amplification, expression of its late genes and ultimately whole virus assembly are conducted in differentiated suprabasal epithelial cells. HPVs’ genes E6 and E7 prevent the infected cells from exiting the cell cycle by targeting p53 and RB for proteasome-dependent degradation, thus allowing them to enter S phase after bypassing G1 checkpoint control [44].

HPVs are involved in several human cancers, and the first association has been observed in the 1970s [127,128,129]. Following characterization of viral subtypes, HPV16 and HPV18 were identified in cervical carcinoma [130], lately followed by the demonstration of their causal role [131]. Overall, HPV in the United States causes about 3% of overall cancers in women and about 2% of overall cancers in men. This percentage increases for certain cancers, namely cancers of the anogenital tract, penis, vulva, vagina, anus, oropharynx (>50% of cancers in females, about 5% in males) [131]. Importantly, while transient HPV infections affects the majority of young women, chronic infection with “high-risk” HPV genotypes, such as types 16 and 18, increases the risk that precancerous lesions may progress to invasive cancer [132], and it is responsible for over two-thirds of cervical cancers [44,133].

HPV can cause genetic changes to the host genome that can initiate and contribute to carcinogenesis, and the mechanisms responsible for these effects have been extensively studied and some of them have been characterized in detail. To this regard, the oncogenic properties of the high-risk HPVs (a total of 12 HPVs are believed to high risk including HPV16, -18, and -31, -33, -35, -39, -45, -51, -52, -56, -56, -59) are believed to be linked mostly to the proteins E6 and E7 [134,135,136,137,138].

#### Effect of HPV on DNA Repair

A number of cellular proteins involved in DNA repairs pathways are affected by HPV proteins. For example, the activation of the ATM pathway in undifferentiated human keratinocyte cell lines is necessary for viral genome amplification in the suprabasal layer, while it is not required for viral episomal maintenance [57].

Consistent with ATM activation, MRN, p-ATM, p-CHK2, and γH2AX all accumulate within nuclear foci that are reminiscent of sites of DNA damage in both undifferentiated and differentiated HFK-31 cells [57]. Indeed, HPV E7 through its LXCXE motif directly binds to ATM in differentiated cells, thus promoting both activation of CHK2 and a low level of caspase activation, important for cleavage of the HPV E1 replication protein [57]. Finally, the shift from viral genome maintenance to the amplification stage has been linked to a transition among different DNA replication processes, from bidirectional to unidirectional, then to rolling-circle stage and eventually formation of multiple, continuous linear viral genome copies [139].

In another example, HPV16 E2 interacts and co-localizes at centrosomes in mitosis with TOPBP1, an ATR activator, potentially affecting ATR damage signaling directly in either undifferentiated or differentiated cells infected with HPV [140]. The fact that HPV16E1/E2-mediated DNA replication occurs in the presence of the topoisomerase inhibitor etoposide, which activates both the ATM and ATR pathways, seems indeed to implicate these two pathways in viral replication [141]. Finally, other two HPV proteins, E1 and E2, co-localize with ATM, ATRIP, MRN, Ku70/86, CHK2, and CHK1 at integrated HPV18 genome replication centers, leading to the activation of DDR pathway [60].

Taken together, these data further suggest that expression of certain HPV proteins selectively target and activate DDR proteins in the ATM and ATR pathways to facilitate production of viral DNA during infection.

Moreover, E6 and E7 can induce DNA damage and promote γH2AX focus formation [61]. In particular, HPV16 E7 appears to accelerate proteo-lytic turnover of the ATR activator claspin, thus promoting mitotic entry in the presence of DNA damage and it also activates DDR pathways [62,63]. Given that HPV genome into the host genome is frequently observed in HPV-mediated tumorigenesis, it could be speculated that direct deregulation by the E6 and E7 proteins from ‘high-risk’ HPV of DDR and DSBR pathways leading to host-cell genomic instability could promote DNA DSBs generation and/or repair, eventually facilitating integration of the viral genome. Indeed, blocking expression of the Ku70 regulatory subunit of DNA-PK, important for both DDR and DSBR, results in loss of episomal HPV16 genomes and reduced integration of HPV16 into the host genome [142].

Another important pathway involved in DNA repair is the Fanconi Anemia (FA) pathway [64]. Not surprisingly, E7 from ‘high-risk’ HPV types might activate the FA pathway and indeed cervical carcinoma tissue exhibits enhanced nuclear FANCD2 focus formation, and this effect is enhanced by the E6 protein. On the other hand, E7 from ‘low risk’ HPW do not show the same property [62]. Both RB-dependent and -independent mechanisms have been proposed to explain how HPV16 E7 activates the FA pathway, and indeed HPV16 E7 expression in FA-deficient cells increases chromosomal instability and apoptosis [62,65]. Finally, a recent study showed a direct association between HPV, the FA pathway and SCC tumor susceptibility [143], which may help to explain why FA patients with HPV have increased susceptibility to squamous cell carcinoma (SCC) [144].

### 3.3. HBV and HCV

The two hepatitis viruses, hepatitis B virus (HBV) and hepatitis C virus (HCV), although similar in name, are different both in genomic composition and structure. The genome of HBV, a member of the Hepadnaviridae family, is about 3.2 kb long, partially dsDNA. HCV has a small RNA genome (about 9.6 kb) which encodes for 10 proteins. HBV is transmitted through the bodily fluids of an infected person, including blood, sweat, tears, saliva, semen, vaginal secretions, menstrual blood, and breast milk. HCV is mainly transmitted through blood-to-blood contact. Both viruses are responsible for chronic liver infections, liver failure, cirrhosis, and hepatocellular carcinoma (HCC) [145,146].

#### Effect of HBC and HCV on DNA Repair

Most of the transforming activities of HBV on hepatocytes result from the oncoprotein HBX and its effects on cellular components of the repair pathways [147] (Table 1). In particular, the relationship between HBX and DDR involves its ability to increase damage to DNA in infected cells, most likely by hampering the activity of various protein components of the DDR itself. To this regard, HBX directly binds DDB1 (ZAP-1/UVDDR) in a structurally conserved α-helical region situated between aa 88 and 100 [66] and this leads to severely reduced nucleotide excision–repair activity (NER) [67,68,69,70]. Of note, this binding is also crucial for viral replication and productive infection [148]. HBX also binds to DDB2, leading to stabilization of the viral protein and inducing its nuclear accumulation [149,150,151], though its effect on NER is not fully understood. HBX also reduces NER activity by directly interacting with some proteins of the TFIIH nucleotide basal excision repair complex [71,72,74,75,152], and this may downregulate the expression levels of both XPB and XPD [72]. As a consequence of reduced NER repair function by HBX, cells become hypersensitive to UV irradiation [71,74,153], with increased likelihood of transformation. Finally, following HBV infection, HBX directly binds to p53 and inhibits p53 anticancer functions [76,77,78,79], while also blocking its association with transcription factors belonging to the DDRs, like ERCC3/XPD and ERCC2/XPB [73,78]. Moreover, it has been shown that HBV infection reduces the protein level of Mre11 thus causing genome instability [80]. Finally, Ko and collaborators demonstrated that HBV viral DNA contains sequences motifs that bind to PARP-1 hampering its DNA repair activity, and this may increase the replication efficiency of HBV and promote the development of HCC [81].

HCC is considered one of the most lethal human malignancies, mainly because it is difficult to detect early, it is chemo- and radio-resistant, and it shows active angiogenesis and metastasis. These features are associated with fast recurrence and reduced survival. Several risk factors are associated with cancer development, including hepatitis B and C virus infection, and genetic alterations together with genomic instability are progressively more accepted as a common feature of human HCC. In this regard, the mechanisms of carcinogenesis induced by HCV are not well defined, but two proteins are believed to be mainly involved, namely the core protein and NS3. HCV core protein has been demonstrated to bind directly (or indirectly interact) with several transcription factors, including hnRNPK, LZIP, RNA helicase CAP-Rf, p53, p21, DDX3 protein, NF-κB, and 14-3-3 protein. It has been observed an association between the development of hepatocellular carcinoma and HCV infection, which also increases cells’ sensitivity to ionizing radiation and bleomycin, a molecule that induces DSBs, while at the same time inhibiting nonhomologous end-joining repair. In addition, the viral core and NS3 proteins hampered DNA repair following damage caused by nitric oxide and reactive oxygen species. Accordingly, stable expression both in vitro and in vivo of core protein caused recurrent chromosome translocations in cultured cells and in transgenic mice, respectively. Indeed, it has been demonstrated that HCV core protein binds to the NBS1 protein and inhibits the formation of the Mre11-NBS1-Rad50 complex, thus hampering ATM activation and inhibiting proper DNA binding of critical enzymes involved in repair pathways. Another protein expressed by HCV, namely Nonstructural Protein 5A (NSP5A), has been correlated to HCV-mediated reduction of DNA-repair related mechanisms. In fact, it was shown that binding of NSP5A to RAD51-associated protein 1 (RAD51AP1), a protein involved in homologous recombination and DNA repair, increased its level through modulation of the ubiquitin-proteasome pathway. This eventually resulted in reduced activity of the RAD51/RAD51AP1/UAF1 complex and consequently increased sensitivity to DNA damage of HCV-infected cells [83]. Finally, inducible expression of hepatitis C virus proteins (UHCV57.3) in recombinant cell culture-derived hepatitis C virus (HCVcc) was used to study the induction of viral proteins in the presence of overexpressed PP2Ac (protein phosphatase 2A). This resulted in inhibition of histone H4 methylation/acetylation and histone H2AX phosphorylation and inhibition of DNA damage repair functions [84]. These data show that upon infection, HCV hinders several DNA repair pathways eventually leading to chromosome instability [82].

### 3.4. EBV (HHV-4) and KSHV (HHV-8)

Epstein–Barr Virus (EBV), also called human herpesvirus 4 (HHV-4), was first observed in Burkitt’s lymphoma cells by electron microscopy [154]. Soon thereafter, with the discovery that more than 90% of people are infected by EBV in their youth, it was documented the widespread presence of EBV and the almost ubiquitous diffusion EBV infections [155]. Though the link between EBV and cancer has been confirmed [156,157], and a number of mechanisms of cellular transformation have been described (Table 1), EBV is a very poor and inefficient carcinogenic agent. Similarly, widespread and with similarly poor transforming abilities is Kaposi’s sarcoma-associated herpesvirus (KSHV), or human herpesvirus 8 (HHV-8) [158]. HHV-8 is a DNA virus belonging to the gamma herpes virus family, codifies for several proteins able to mediate cellular transformation (Table 1). It is present in a significant amount of the population, though not as prevalent as EBV and it rarely causes cancer except when in the presence of HIV, which obviously acts as a cofactor. However, its widespread presence makes its contribution to cancer significant.

#### Effect of EBV (HHV-4) and KSHV (HHV-8) on DNA Repair

Both EBV and KSHV interaction with certain cellular proteins eventually results in reduced activity of the DDR pathways during both the latent phase and the lytic infection. For example, KSHV v-cyclin, expressed in both phases, activates the DDR, as demonstrated by phosphorylation of H2AX, CHK2 and p53, and induces S-phase arrest [96]. Among the proteins more specifically expressed in the case of latent infection, EBNA-1, which has been demonstrated to promote the generation of reactive oxygen species that cause DNA damage. This would activate DNA-repair pathways that are, in turn, hampered by other viral proteins [85,86]. To this regard, we highlight the effect of LMP1 which downregulates ATM, eventually resulting in CHK2 phosphorylation and abrogation of G2 checkpoint. In addition, LMP1 blocks DNA repair by activation of the PI3K/Akt pathway that results in reduced activity of FOXO3a [87].

In the case of the lytic phase, there seems to be different proteins involved in deregulating DDR. First, we note the induction of an ATM-dependent DDR [88]. Indeed, the recruitment of the MRN complex, p-p53 and p-ATM, to VRCs suggests that the cell recognizes damaged linear viral DNA as damaged cellular DNA [88]. By inactivation of p53, EBV is thus able to evade ATM-mediated checkpoints, holding the host cell in S phase and thus facilitating virus replication [88]. Moreover, the IE lytic transactivator BZLF1 of EBV recruits Cul2- and/or Cul5-CRLs, to p53 which in turn causes its ubiquitylation and proteasome-mediated degradation [89] independently of Mdm2 [159]. Additionally, genomic instability in human epithelial cells was induced by the early lytic viral protein BGLF5, a DNase, by direct damaging the cellular DNA, consequently hampering the expression of a number of DNA-repair genes [90]. BGLF5 also contributes to the production of linear viral genomes [91,92] and for this reason it has been hypothesized that it could promote genomic instability during lytic replication. Further, some experimental data indicate that cellular hyper proliferation is responsible for the activation of DDR in cells infected by EBV, and as such is independent of viral replication [93]. Indeed, another mechanism that EBV employs to interfere with cellular damage response is through EBNA-3C, a member of the EBNA proteins family, which has been shown to inhibit DDR in normally proliferating lymphoblastoid cell lines [93].

EBV infection reduces apoptosis induced following DNA damage in Burkitt’s lymphoma-derived B-cells through expression of EBNA-3A and EBNA-3C, which in turn reduce BIM and NOXA expression [94]. These data would indicate that at least in this case the virus can selectively interfere with some aspects of the cellular damage–response pathways, but not with those associated with apoptosis, in order to promote survival and propagation of cells carrying damaged DNA molecules.

EBNA-LP interacts with and seems to be a substrate for DNA-PK. Moreover, it also co-immunoprecipitates with HA95. These are two important proteins of the DDR complex, and these interactions are likely to play a role in the transforming abilities of the virus [95].

## 4. Bacteria–Host Interaction and Effects on Host’s DNA Repair

A number of bacteria have been associated with human cancers, but *Helicobacter pylori* so far is the only one with clear epidemiological data to support causality [160]. Molecular mechanisms employed by these bacteria to alter cellular pathways are still largely unknown, although it is becoming increasingly clear that they can enhance accumulation of DNA-damage and inhibition of p53-activities which play a critical role in driving cellular transformation. As outlined earlier in this review, mammalian cells engage complex mechanisms assuring surveillance of proper genome integrity and repair in case of necessity. These functions are exerted by the so-called DDR pathways, and their reduced activity or failure results in DNA damage accumulation and genomic instability. Data reported by several groups indicate that host cells DNA undergo damage upon bacterial infections, either directly through toxins, or indirectly following activation of the cell’s immune response against the pathogen (Table 2).

Recently, a number of studies have elucidated the role of *H. pylori* infection in causing DSBs in host cells, which eventually results in genomic instability, including microsatellite instability (MSI), chromosomal instability (CIN), and abnormal activation of telomerase [181,182]. Interestingly, these DSBs are preferentially repaired through the NHEJ pathways rather than the HR one, suggesting upregulation of NHEJ-related genes and downregulation of HR-related ones. In a study assessing the expression of 179 genes implicated in various DDRs pathways in uninfected and infected cells, 58 genes were downregulated more than two-fold, including NBS1, ATR, MLH1, and TP53, while only 11 genes were upregulated, further suggesting that *H. pylori* infection causes a systematic reduction in DNA repair capacities [161].

Free radicals produced by immune cells in response to infections, but also made by infected cells themselves, may cause the formation of oxidized bases as well as apurinic and apyrimidinic sites (AP sites, also known as abasic sites), and strand breaks. For several bacterial pathogens, the mechanisms through which these bacteria contribute to the production of intracellular ROS have been described [183,184]. In particular, *H. pylori* infection results in ROS and RNS production leading to a number of *H. pylori*-related gastric diseases in several animal models and human studies [162,163]. *H. pylori*-mediated inflammation has been shown in gastric cancer indicating that *H. pylori* infection induces oxidative DNA damage leading to DSBs, which in turn activates the DNA damage response pathway in gastric epithelial cells [185]. Failure to repair this DNA damage by one of the several DDR pathways has the potential to accumulate mutations that can eventually lead to cellular transformation [186,187].

On the other hand, certain bacterial pathogens can also induce DSBs with a mechanism unrelated to ROS production by hijacking other cellular responses [164,188,189,190]. Indeed, *H. pylori* infection was associated with increased γH2AX expression (a biomarker of DNA double-strand breaks), as assessed by immunohistochemical staining of γH2AX, and γH2AX was found to correlate with a number of clinicopathological characteristics in GC tissues infected by *H. pylori*. This suggests that DSBs appear to be an early molecular event in gastric carcinogenesis associated with *H. pylori* infection [164]. Another potential mechanism is through interaction of its type IV secretion system protein complex with host cell integrin β1, eventually resulting in NF-κB activation and subsequent recruitment of the endonucleases xeroderma pigmentosum group G-complementing protein (XPG, also known as ERCC5) and xeroderma pigmentosum group F (XPF, also known as ERCC4) to host cell chromatin [165,166]. In other cases, DSB induction by the Cag pathogenicity island (CagPAI)-positive strains of *H. pylori* seems to be mediated by NF-κB activation leading to aberrant expression of activation-induced cytidine deaminase AID [167,168].

Another example of ROS-independent DSB generation is observed upon *Listeria monocytogenes*-infection, where cell cycle progression is delayed in S phase, but not completely arrested, thus facilitating bacterial replication. It is thus conceivable that, following infection, the bacterium causes nucleotide pool depletion to support its own replication and growth, leading to replication fork stalling and, consequently, DNA breaks [169].

Upon infection, some intestinal bacteria produce toxins that cause DNA lesions [170], potentially resulting in genome instability, and tumor initiation and progression. At the moment, four genotoxins are known to have this effect, namely colibactin (expressed by *Escherichia coli*) [171], cytolethal distending toxin (CDT; expressed by certain Gram-negative bacteria [172], Shiga toxin (expressed by *Shigella dysenteriae*) [173,174,175], and endonucleases (expressed by *Neisseria gonorrhoeae*) [176,177,178].

Finally, several species of Mycoplasmas have been associated with human cancers [191,192,193], and the ability of certain strains to reduce p53 activity has been proposed as a prominent mechanism for cellular transformation [194,195]. We recently demonstrated that DnaK, a chaperon protein belonging to the HSP70 family from a subspecies of *Mycoplasma fermentans*, hampered PARylation activity of PARP1 upon DNA damage [179,180]. PARP 1 is arguably the most studied component of the PARP proteins family [196], which acts by modifying (PARylating) and activating certain proteins involved in recognition and repair of single and double-strand DNA breaks [197,198]. Upon interaction with forms of damaged DNA, PARP1 increases its activity, leading to PARylation of specific proteins, including, among others, PARP1 itself, DNA-dependent protein kinase (DNA-PK), topoisomerase 1 (TOP1), histones. This in turn results in recruitment of single-strand break repair (SSBR)/base-excision repair (BER) factors to the damaged site [199,200]. Inability to accurately repair DNA damage ordinarily leads to apoptosis, in order to avoid accumulation of DNA damage that could eventually result in cellular transformation.

Our results showed decreased PARylation of proteins between 100–150 KDa in size in cells transfected with a vector expressing *M. fermentans* DnaK, as opposed to cells transfected with control, empty vector [179]. We also showed that Mycoplasma DnaK, co-immunoprecipitates with USP10, a key p53 regulator, thus impairing p53-dependent anti-cancer functions, resulting in reduced efficacy of anti-cancer drugs that depend on p53 activation to exert their effect [180]. Phylogenetic amino acid analysis shows that other bacteria associated with human cancers (including certain Mycoplasmas, *Helicobacter pylori*, *Fusobacterium nucleatum*, and *Chlamydia thrachomatis*) have highly related DnaKs, suggesting a possible common mechanism of cellular transformation [180]. Our data thus indicate that mycoplasmas, and perhaps certain other bacteria with closely related DnaK, may alter DNA repair mechanism and anti-cancer drug response and therapy activity mediated through inhibition of p53 functions.

## 5. Conclusions

**Figure 1:**
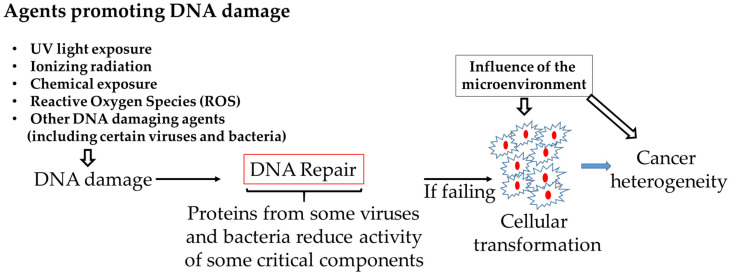
Proteins from some viruses and bacteria may promote cellular transformation by reducing the activity of cellular pathways involved in DNA repair.

A large amount of DNA damage occurs per cell every day due to numerous causes, including viral and bacterial infections. The majority of them are repaired, but the repair processes are not very efficient due to interferences of the bacterial and viral proteins with the host cellular machineries. Unrepaired damage increases mutations, activating oncogenes or inactivating tumor suppressor genes that ultimately cause genomic instability thereby increasing the risk of cancer. By interfering with pathways responsible for DNA repair, resulting in accumulation of mutations and a consequent greatly increased chance of cellular transformation, the proteins of certain viruses or bacteria would thus similarly contribute to cellular transformation. The tumor microenvironment could then affect tumor cell growth [201] (Figure 1). Our review provides a framework to better understanding the common role played by some viruses and bacterial proteins in disrupting the role of cellular proteins implicated in DNA damage and repair, eventually leading to cellular transformation. This knowledge would also have implications for the prevention and treatment of cancer.

## Figures and Tables

**Table 1:** Viruses and DNA damage.

Virus	Proteins Involved in DNA Damage	Mechanisms
**Human T-cell leukemia virus type1 (HTLV-1)**	Tax p30 HBZ	- Tax expressing cells have reduced levels of Ku80 mRNA [45]; - Tax constitutively activates DNA-PK and attenuates ATM signaling in response to DNA damage [46,47]; - Tax binds and sequesters other factors in damage-independent nuclear foci, including BRCA1, and MDC1 [48]; - Tax induces DNA DSBs during DNA replication, thus promoting genetic instability while also activating the NF-κB pathway to eventually inhibit the HR DNA repair pathway [49]; - Tax alters the levels of key cellular factors such as DNA polymerase β and PCNA [50,51,52,53]; - Tax inactivates transcription of telomerase, and this in turn can leave unprotected DNA ends that are more susceptible to recombination and translocation events, in turn leading to karyotypic abnormalities [54]; - p30 inhibits the conservative HR DNA repair by targeting the MRE11/RAD50/NBS1 complex, and that in turn promotes the error-prone NHEJ DNA-repair pathway [55]; - HBZ interacts with Ku70 and Ku80 and reduces repairs of DNA DSBs via NHEJ [56].
**Human papillomavirus (HPV)**	E1 E2 E7	- E7 through its LXCXE motif directly binds to ATM, thus promoting both activation of CHK2 and a low level of caspase activation [57]; - HPV16 E2 interacts and co-localizes at centrosomes in mitosis with TOPBP1, affecting ATR damage signaling directly [58,59]; - E1 and E2 co-localize with ATM, ATRIP, MRN, Ku70/86, CHK2 and CHK1 at integrated HPV18 genome replication centers, leading to the activation of DDR pathway [60]; - E6 and E7 can induce DNA damage and promote γH2AX focus formation [61]; - HPV16 E7 appears to accelerate proteo-lytic turnover of the ATR activator claspin, thus promoting mitotic entry in the presence of DNA damage and it also activates DDR pathways [62,63]; - E7 from “high-risk” HPV types might activate the FA pathway and indeed cervical carcinoma tissue exhibits enhanced nuclear FANCD2 focus formation, and this effect is enhanced by the E6 protein [64]; - HPV16 E7 expression in FA-deficient cells increases chromosomal instability and apoptosis [62,65].
**Hepatitis B Virus (HBV)**	HBX	- HBX directly binds DDB1 [66] and this leads to severely reduced NER [67,68,69,70]; - HBX reduces NER activity by directly interacting with some proteins of the TFIIH nucleotide basal excision repair complex [71,72,73,74,75], and this may downregulate the expression levels of both XPB and XPD [72]; - HBX directly binds to p53 and inhibits its anticancer functions [76,77,78,79], while also blocking its association with transcription factors belonging to the DDRs, like ERCC3/XPD and ERCC2/XPB [73,78]; - HBV infection reduces the protein level of Mre11 thus causing genome instability [80]; - HBV viral DNA contains sequences motifs that bind to PARP-1 hampering its DNA repair activity, and this may increase the replication efficiency of HBV and promote the development of HCC [81].
**Hepatitis C virus (HCV)**	Core protein NSP5A UHCV57.3	- HCV core protein binds to the NBS1 protein and inhibits the formation of the Mre11-NBS1-Rad50 complex, thus hampering ATM activation and inhibiting proper DNA binding of critical enzymes involved in repair pathways [82]; - NSP5A binds to RAD51AP1 and this eventually results in reduced activity of the RAD51/RAD51AP1/UAF1 complex and consequently increased sensitivity to DNA damage of HCV-infected cells [83]; - Inducible expression of UHCV57.3 results in inhibition of histone H4 methylation/acetylation and H2AX phosphorylation and inhibition of DNA damage repair functions [84].
**Epstein–Barr virus (EBV)**	EBNA-1 LMP-1 BZLF1 BGLF5 EBNA-3C EBNA-LP	- EBNA-1 promotes the generation of ROS that cause DNA damage [85,86]; - LMP-1 downregulates ATM, eventually resulting in CHK2 phosphorylation and abrogation of G2 checkpoint. LMP-1 blocks also DNA repair by activating the PI3K/Akt pathway that results in reduced activity of FOXO3a [87]; - EBV inactivates p53, leading to ATM-mediated checkpoints evasion, holding the host cell in S phase and thus facilitating virus replication [88]; - The IE lytic transactivator BZLF1 recruits Cul2- and/or Cul5- CRLs, to induce p53 degradation [89]; - Early lytic viral protein BGLF5 directly damages the cellular DNA and consequently hampers the expression of several DNA-repair genes [90]; - BGLF5 contributes to the production of linear viral genomes and it promotes genomic instability during lytic replication. [91,92]; - EBNA-3C inhibits DDR in normally proliferating lymphoblastoid cell lines [93]; - EBV infection reduces apoptosis induced following DNA damage in Burkitt’s lymphoma-derived B-cells through expression of EBNA-3A and EBNA-3C, which in turn reduce BIM and NOXA expression [94]; - EBNA-LP interacts with DNA-PK and co-immunoprecipitates with HA95 [95].
**Kaposi’s sarcoma-associated herpesvirus (KSHV) or Human herpesvirus 8 (HHV-8)**	V-cyclin	- V-cyclin activates the DDR by phosphorylating H2AX, CHK2 and p53, and inducing S-phase arrest [96].

The first column lists the names of the viruses in bold.

**Table 2:** Bacteria and DNA damage.

Bacteria	Effects on Host’s DNA Repair
*Helicobacter pylori*	The infection causes a systematic reduction in DNA repair capacities by downregulating 58 genes more than two-fold (such as NBS1, ATR, MLH1, and TP53) [161]; The infection results in ROS and RNS production leading to a number of gastric diseases [162,163]; The infection is associated with increased γH2AX expression, and γH2AX was found to correlate with a number of clinicopathological characteristics in GC tissues infected by *H. pylori* [164]; Type IV secretion system protein complex interacts with host cell integrin β1, eventually resulting in NF-κB activation and subsequent recruitment of XPG and XPF [165,166]; Cag mediates NF-κB activation leading to aberrant expression of activation-induced cytidine deaminase AID [167,168].
*Listeria monocytogenes*	It causes nucleotide pool depletion to support its own replication and growth, leading to replication fork stalling and, consequently, DNA breaks [169].
*Escherichia coli*, Gram-negative bacteria, *Shigella dysenteriae* and *Neisseria gonorrhoeae*	They all produce toxins that cause DNA lesions [170], potentially resulting in genome instability, tumor initiation and progression: Colibactin (*E. coli*) [171], Cytolethal distending toxin (CDT) (Gram-negative bacteria) [172], Shiga toxin (*S. dysenteriae*) [173,174,175] Endonucleases (*N. gonorrhoeae*) [176,177,178].
*Mycoplasma fermentans*	DnaK hampered PARylation activity of PARP1 upon DNA damage [179,180]; DnaK co-immunoprecipitates with USP10, thus impairing p53-dependent anti-cancer functions, resulting in reduced efficacy of anti-cancer drugs that depend on p53 activation to exert their effect [180].

## Abbreviations

DDR	DNA Damage Response
NER	Nucleotide excision repair
MMR	Mismatch repair
HR	Homologous recombination
NHEJ	Non-homologous end joining
DSB	Double-strand breaks
BER	Base excision repair
SSB	Single-strand break
ICB	Immune Checkpoint Blockade
